# Potent *in vitro* antiviral activity of *Cistus incanus* extract against HIV and Filoviruses targets viral envelope proteins

**DOI:** 10.1038/srep20394

**Published:** 2016-02-02

**Authors:** Stephanie Rebensburg, Markus Helfer, Martha Schneider, Herwig Koppensteiner, Josef Eberle, Michael Schindler, Lutz Gürtler, Ruth Brack-Werner

**Affiliations:** 1 Helmholtz Zentrum München - Deutsches Forschungszentrum für Gesundheit und Umwelt, Institute of Virology, Oberschleißheim; 2 University Hospital of Tübingen, Department of Medical Virology and Epidemiology of Viral Diseases, Tübingen; 3 Ludwig Maximilian’s University, Max von Pettenkofer Institute, Munich.; 4 German Center for Infection Research, partner site Munich, Germany.

## Abstract

Novel therapeutic options are urgently needed to improve global treatment of virus infections. Herbal products with confirmed clinical safety features are attractive starting material for the identification of new antiviral activities. Here we demonstrate that *Cistus incanus* (Ci) herbal products inhibit human immunodeficiency virus (HIV) infections *in vitro*. Ci extract inhibited clinical HIV-1 and HIV-2 isolates, and, importantly, a virus isolate with multiple drug resistances, confirming broad anti-HIV activity. Antiviral activity was highly selective for virus particles, preventing primary attachment of the virus to the cell surface and viral envelope proteins from binding to heparin. Bioassay-guided fractionation indicated that Ci extract contains numerous antiviral compounds and therefore has favorably low propensity to induce virus resistance. Indeed, no resistant viruses emerged during 24 weeks of continuous propagation of the virus in the presence of Ci extracts. Finally, Ci extracts also inhibited infection by virus particles pseudotyped with Ebola and Marburg virus envelope proteins, indicating that antiviral activity of Ci extract extends to emerging viral pathogens. These results demonstrate that Ci extracts show potent and broad *in vitro* antiviral activity against viruses that cause life-threatening diseases in humans and are promising sources of agents that target virus particles.

Virus infections pose serious threats to human health on a global scale. The human immunodeficiency virus (HIV) causes chronic, life-long infection that leads to AIDS without antiretroviral treatment. Over 35 million individuals are living with HIV/AIDS and the pandemic has remained among the 10 leading global causes of death for over a decade, especially in low-income countries^1^. Emerging viruses like the Ebola virus (EBOV) can cause an acute disease with symptoms appearing 2–21 days after infection and a high risk of death (average mortality rate ~50%) (For summary of current knowledge regarding Ebola virus disease see^2^). EBOV can cause sudden outbreaks, like the one that flared up in May 2014 in West Africa (Guinea, Liberia and Sierra Leone). Next to the death of more than 10 000 individuals, this outbreak placed an additional heavy burden on countries already weakened by lack of resources and long periods of conflict and instability and lead to breakdown of local health care systems.

There is a strong need for the development of novel antiviral agents for treatment of life-threatening viral infections. Despite the availability of a number of approved drugs for treatment of HIV/AIDS (≥25^3^), current anti-HIV therapies would still benefit from various improvements. Limitations include the high risk of emergence of resistant viruses, poor penetration of virus sanctuaries like the central nervous system, adverse effects, especially in the context of long-term therapy, and incomplete access to affordable therapies in resource-limited areas^4^,^5^,^6^,^7^. Furthermore, the majority of these drugs block post-entry steps of the virus replication cycle and attack only a few viral targets, such as the viral reverse transcriptase, protease or integrase^3^.

In contrast to HIV/AIDS, no approved drugs are currently available to combat EBOV infections. The lack of targeted antiviral therapies is one of the most frightening aspects of managing EBOV outbreaks.

Plant-derived natural products play a significant role for medical treatments^8^,^9^. Herbal extracts represent the primary form of health care for a major proportion of the world’s population^10^ and are an important source of single-molecule drug leads. A prominent example is the anti-malaria activity of *Artimisia annua*^11^ discovered by YouYou Tu, recipient of the 2015 Nobel Prize for Physiology and Medicine^12^.

Antiviral activities have been reported for numerous medicinal plants^9^,^13^. However, their implementation as herbal antiviral medicines requires in-depth research of their efficacy, safety, composition and mechanisms-of-action^14^. Data for clinical safety and for biological activities are available for a few herbal extracts, some of which are sold as over-the-counter medicines in Europe. We seek to investigate the antiviral activities of these clinically evaluated herbal medicines against major human viral pathogens like HIV-1 as an initial step for the identification of new sources of antiviral agents. This approach is supported by our recent demonstration of anti-HIV-1 activity of extracts of *Pelargonium sidoides*^15^, licensed in Germany as the herbal medicine Umckaloabo®.

In this study, we investigated antiviral activities of *Cistus incanus* (Pink Rockrose) against HIV and Filoviruses. *Cistus incanus* (Ci) is native to Mediterranean regions of Southern Europe and North Africa and belongs to a different taxonomic order (Malvales) than *Pelargonium sidoides* (Geraniales). Ci extracts have been shown to have anti-inflammatory, anti-oxidant, antimycotic and antibacterial activities^16^,^17^,^18^,^19^. Ci is rich in polyphenols^18^,^19^,^20^,^21^,^22^,^23^, a chemical class of compounds that includes many representatives with antimicrobial/antiviral activities^24^,^25^. Furthermore, Ci extracts were demonstrated to inhibit infection by influenza A virus^21^,^26^. Different Ci preparations are commercially available, including a CYSTUS052^®^ decoction, throat lozenges and a herbal tea. Clinical studies performed with patients with upper respiratory tract infections revealed decreased symptoms and less adverse effects in treated patients compared to control patients, indicating clinical efficacy and a favorable safety profile of CYSTUS052^®^^27^,^28^.

We demonstrate that Ci extracts show broad inhibitory activity against different HIV isolates, including a clinical virus isolate with multiple resistances against conventional drugs. Mode-of-action studies demonstrate that Ci extracts target viral envelope proteins, preventing the primary attachment of the virus to host cells. Antiviral activity of Ci extracts was also directed against the Ebola virus envelope protein. Extract deconvolution studies revealed that Ci extracts contain numerous active ingredients against HIV and Ebola virus. Our results demonstrate that Ci extract has potent antiviral activity against HIV and Ebola virus and indicate that Ci extract contains multiple compounds that prevent these viruses from entering host cells for replication.

## Results

### Antiviral activity of *Cistus incanus* extracts against a broad range of HIV isolates

Different aqueous extracts of *Cistus incanus* (Ci) were evaluated for anti-HIV activity, using a highly sensitive HIV reporter cell line (LC5-RIC) and technology established for identification of HIV inhibitors (EASY-HIT^29^). Initial testing was performed with a commercial Ci preparation (CYSTUS052®), an extract brewed from Ci tea and an extract prepared from fresh, self-grown Ci plants (n = 3). All three Ci extracts inhibited infection of LC5-RIC cells by the HIV-1 laboratory isolate HIV-1_LAI_ with similar efficacies (Supplementary Fig. S1). Viability of Ci treated cells (measured by MTT assay) was ≥80% of untreated cells for all extracts. For further analysis of anti-HIV activity, we used CYSTUS052®.

**Figure 1:**
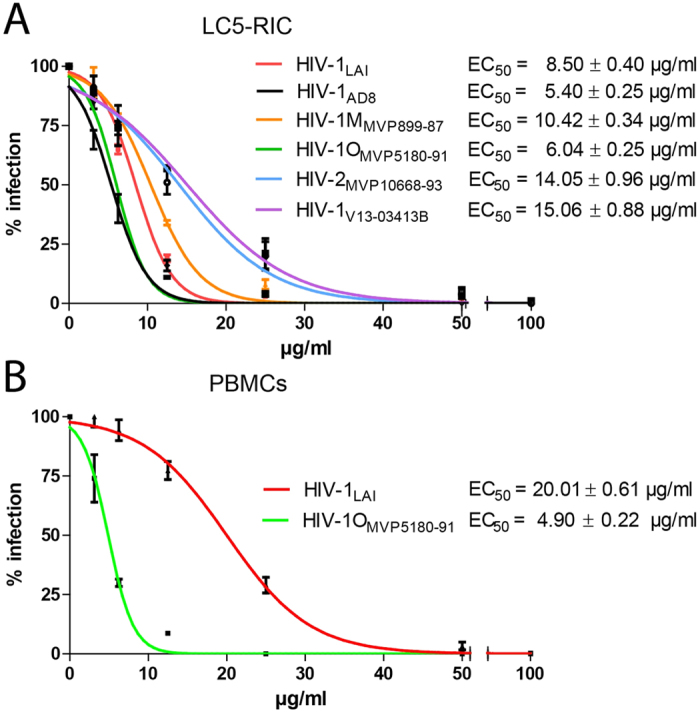
*Cistus incanus* extract inhibits infectivity of a broad range of human immunodeficiency viruses (HIV). (**A**) *Antiviral activity of Cistus incanus (Ci) extract inhibits HIV type 1 and HIV type 2 isolates*. Inhibition of infection was evaluated with human HIV reporter cells (LC5-RIC or LC5-RIC-R5 for HIV-1_AD8(R5)_) using HIV-1 isolates with different coreceptor tropisms (HIV-1_LAI_: X4-tropic; HIV-1_AD8(R5)_: R5-tropic), HIV-1 clinical isolates belonging to the M (Major) (HIV-1M_MVP899-87_) or the O (Outlier) group (HIV-1O_MVP5180-91_), a clinical isolate of HIV type 2 (HIV-2_MVP10668-93_ ) and a clinical HIV-1 isolate with multiple drug resistances (HIV-1_V13-03413B_). (**B**) *Cistus incanus (Ci) extract inhibits HIV infection of primary human target cells.* Interleukin-2 stimulated peripheral blood mononuclear cells (PBMC) were exposed to HIV-1_LAI_ or HIV-1O_MVP5180-91_ virus in the presence of different concentrations of Ci extract. Levels of infectious virus produced by the PBMC were determined by analysing culture supernatants of the PBMC with LC5-RIC cells. Experiments were performed with PBMCs from 3 donors. Dose-response curves are shown for 3 independent infection assays, each performed with 6 concentrations of Ci extract between 100 μg/ml and 3.12 μg/ml. Symbols signify mean values and error bars the standard deviation of the mean. EC_50_ signifies the calculated half maximal effective concentration.

We then investigated antiviral activity of the Ci extract against a broad range of clinical HIV isolates. As shown in Fig. 1, Ci extract inhibited infection by both HIV types 1 and 2. Inhibition of infection by clinical isolates from two different groups [Major (HIV-1M_MVP899-87_) and Outlier (HIV-1O_MVP5180-91_)] was shown with LC5-RIC reporter cells and with primary human peripheral blood mononuclear cells (PBMC). Importantly, Ci extract also inhibited an HIV-1 M group/B clade isolate (HIV-1_V13-03413B_) with multiple drug resistance mutations. This isolate was derived from a patient with virologic treatment failure. Analysis of the protease and reverse transcriptase sequences of HIV-1_V13-03413B_ with the HIV-GRADE tool^30^ predicted high-level resistance of HIV-1_V13-03413B_ against 3 of 4 NNRTIs (Non-nucleoside reverse transcriptase inhibitors), 8 of 10 NRTIs (nucleoside reverse transcriptase inhibitors) and 13 of 14 protease inhibitors, and intermediate resistance against the remaining drugs in these classes.

**Figure 2:**
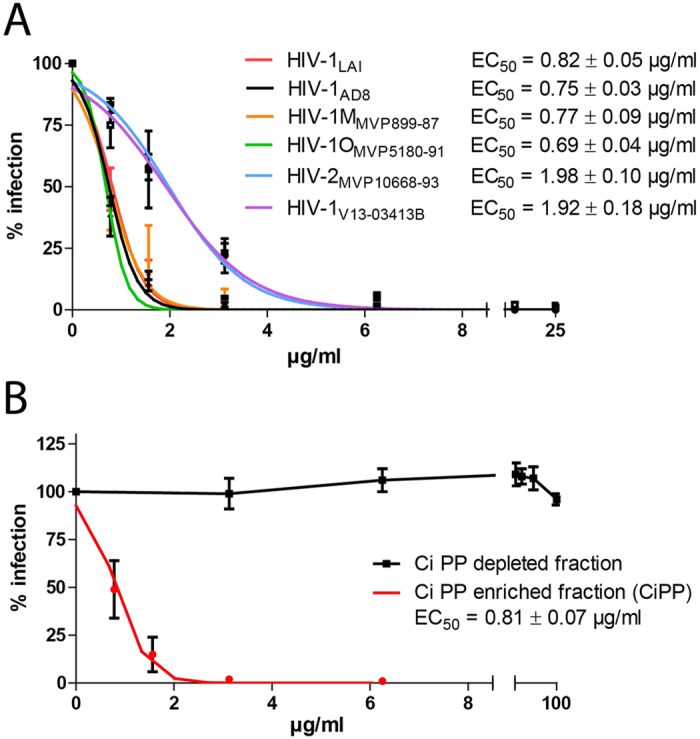
Polyphenols are crucial for anti-HIV activity of the *Cistus incanus* extract. A polyphenol-enriched fraction (CiPP-enriched fraction) was generated by exposing whole *Cistus incanus* (Ci) extract to polyvinylpyrrolidone (PVPP), followed by separation of the PVPP pellet from the aqueous supernatant by centrifugation and elution of the adsorbed polyphenols (PP) from the PVPP pellet. (For further details see Materials and Methods). (**A**) *Polyphenol-enriched fraction efficiently inhibits infection by HIV type 1 and HIV type 2 isolates.* HIV-inhibitory activity of the polyphenol-enriched fraction was tested with the same panel of HIV isolates with LC5-RIC cells and under the same conditions used for testing of whole Ci extract (see Fig. 1). (**B**) *Anti-HIV activity is selectively retained by the polyphenol-enriched fraction*. Anti-HIV activities of the polyphenol-enriched and the polyphenol depleted fraction were compared using HIV-1_LAI_ and LC5-RIC cells. The polyphenol-depleted fraction (=CiPP-depleted fraction) represents the aqueous supernatant of the PVPP pellet after centrifugation. Dose-response curves are shown for 3 independent infection assays, each performed with 6 concentrations of Ci extract between 25 μg/ml and 0.78 μg/ml. Symbols signify mean values and error bars the standard deviation of the mean. EC_50_ signifies the calculated half maximal effective concentration.

To elucidate the importance of polyphenols for anti-HIV activity of Ci extract, we produced a polyphenol-enriched fraction (CiPP) from the Ci extract by adsorption of polyphenols to polyvinylpyrrolidone (PVPP). CiPP showed dose-dependent antiviral activity against the entire panel of HIV isolates tested (Fig. 2A). The antiviral activity of the CiPP fraction ranged from ~0.7 to 2.0 μg/ml and was ≥5-fold stronger than that of the whole Ci extract (compare Figs 1A and 2A). In contrast, the polyphenol-depleted fraction showed no antiviral activity when tested with HIV-1_LAI_ at the same concentrations, indicating that polyphenols account for the antiviral activity of Ci extract (Fig. 2B).

Testing of the effect of Ci treatment on the viability of PBMC by MTT assay revealed CC_50_ values ≥250 μg/ml for the whole extract (Supplementary Fig. S2). CiPP showed even lower cytotoxicity (CC_50_ >1200 μg/ml) than the whole extract. Thus enrichment of polyphenols eliminates cytotoxic components of the extract and increases antiviral activity.

Together these results demonstrate potent antiviral activity of Ci extract against a broad range of HIV isolates and show that polyphenols are crucial for anti-HIV activity.

### Mechanism-of-action: Ci extract prevents HIV from invading host cells

**Figure 3:**
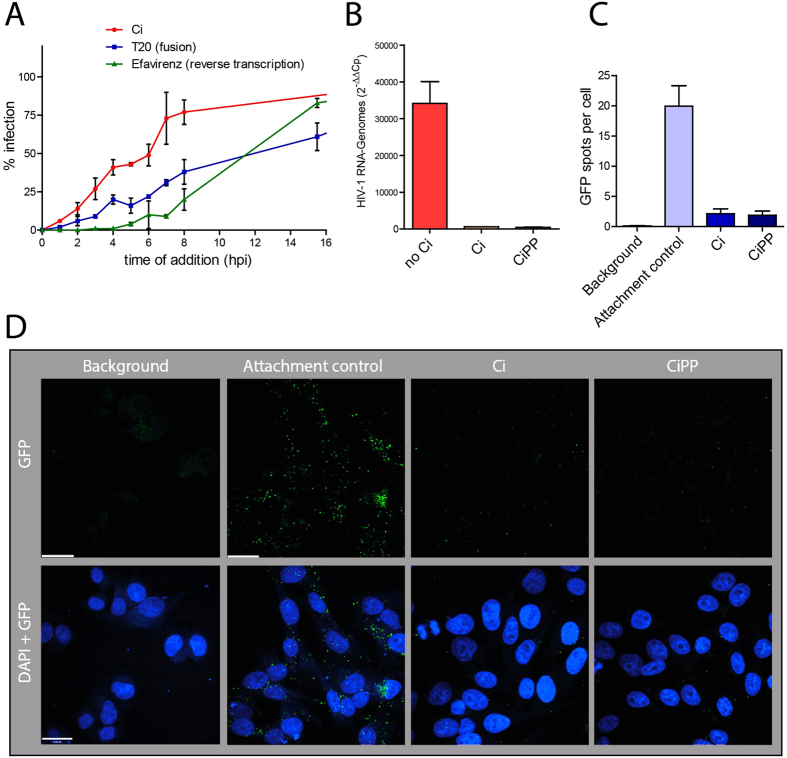
Cistus incanus extract blocks invasion of cells by HIV. (**A**) *Antiviral activity of Ci extract inhibits a very early phase of the HIV replication cycle.* Time-of-addition (TOA) assays were performed with *Cistus incanus* (Ci) extract or the reference inhibitors T20 (virus-cell fusion) and Efavirenz (reverse transcription). Each time point was assayed in triplicate wells and infection levels normalized to those of untreated cultures (=100%). Symbols indicate mean values and error bars standard deviation of the mean. (**B**) *Ci extract prevents delivery of genomic HIV RNA to cell*s. LC5-CD4 cultures were exposed to HIV-1_LAI_ in the absence (no Ci) or in the presence of 100 μg/ml whole Ci extract (Ci) or a Ci-derived polyphenol enriched fraction (CiPP) for 4 hours. Efavirenz was included to block HIV reverse transcription in target cells. RNA was isolated from cultures after 4 hours of virus exposure, cDNA generated and used for quantitative PCR analysis. Levels of HIV-1 sequences were related to RNA polymerase II reference sequences. The data is expressed as relative levels of HIV-RNA genomes in cultures exposed to HIV-1, normalized to background (i.e. no virus). Each bar represents the mean results of 3 virus exposure experiments Error bars indicate the standard deviation of the mean. (**C**,**D**) *Ci extract prevents attachment of virus particles to cells*. Spinning disc confocal microscopy was used to analyse the influence of Ci or CiPP on the attachment of GFP-labelled HIV-1 virus particles to LC5-RIC cells. Cells were exposed to virus for 4 hours. The fusion inhibitor T20 was included to block entry of virus into target cells. Controls consisted of cells exposed to virus without Ci (attachment control) and unexposed cells (background). Cell nuclei were visualized by DAPI staining. (**C**) Quantitative analysis of cell-associated GFP-spots. GFP-signals associated with 60 cells from 4 images were analysed for each sample. The bars represent the mean values of GFP signals per cell and the error bars indicate the standard deviation of the mean. (**D**) Representative images for each sample. Scale bars: 20 μm.

Antiviral testing of Ci extract in the EASY-HIT system revealed inhibition in the first step of the assay, which is characteristic of antiviral agents that affect the early phase of the HIV replication cycle^29^. We therefore performed time-of-addition (TOA) assays. The TOA assay measures the impact of the time point of addition of the antiviral compound on its capacity to inhibit virus infection^29^,^31^. As reference compounds we used the HIV-1 fusion inhibitor T20 and the reverse transcription inhibitor Efavirenz. The resulting Ci profile shows loss-of-inhibition at time points even earlier than observed for the fusion inhibitor T20 (Fig. 3A), demonstrating that Ci inhibits a very early step in the HIV-1 replication cycle. This is further supported by failure to detect cell-associated genomic viral RNA in HIV-exposed cells co-treated with Ci extract and the reverse transcriptase inhibitor Efavirenz (Fig. 3B).

We then assessed the effect of Ci extract on the attachment of virus particles to host cells, using GFP-labelled HIV-1 particles (HIV-1 NL4-3 Gag-iGFP^32^) and spinning disc confocal microscopy, as described previously^15^. Virus attachment was assayed in the presence of T20 to maximize virus-cell association. As expected, numerous green fluorescent, cell-associated dots were visible in the absence of Ci, confirming attachment of virus particles to host cells (Fig. 3C,D Attachment control). In contrast, treatment with Ci extract strongly reduced the number of GFP signals per cell (Ci extract and CiPP), indicating that Ci extract inhibits the association of virus particles with host cells.

Together these results demonstrate that Ci extract blocks attachment of viral particles to cells and thus inhibit viral entry into host cells.

### Antiviral activity of Ci extract targets HIV envelope proteins

**Figure 4:**
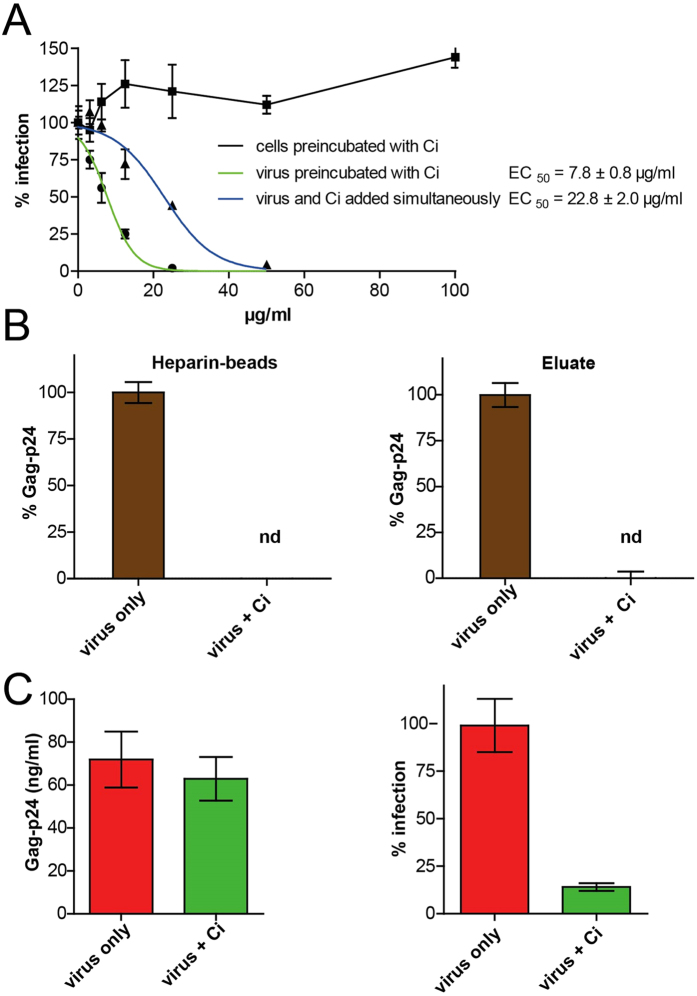
HIV-inhibitory activity of *Cistus incanus* extract selectively targets viral envelope proteins. (**A**) *Antiviral components of Ci extract interact with virus particles but not target cells*. The graph shows the effects of preincubation of virus inoculum (HIV-1_LAI_ ) or target cells (LC5-RIC) with Ci extract on its anti-HIV-1 activity. As control, antiviral activity was assayed under standard conditions, in which virus inoculum and Ci extract were added to cells simultaneously. Symbols signify mean values for each extract dilution analysed in triplicate and error bars the standard deviation of the mean. Values for infection of cultures treated with Ci extract are expressed relative to untreated cultures (=100% infection). (**B**) *Ci treatment blocks binding of virus particles to heparin.* Virus capture assays were performed with heparin beads and virus samples containing HIV-1_LAI_, incubated either with (virus + Ci) or without Ci extract (virus only). Captured virus was quantified in the bead fraction (left panel) and in the eluate (right panel) by Gag-p24 ELISA. Levels of Gag-p24 antigen captured from Ci treated virus samples are expressed relative to Gag-p24 antigen levels captured from untreated control samples (=100%). Results are shown for two independent capture experiments performed with different amounts of virus. Gag-p24 levels were normalized to values measured for the control sample (virus only =100%). Columns signify mean values and error bars the standard deviation of the mean (nd = not detected). (**C**) *Ci treated virus particles are captured by antibodies that bind to cell-derived CD44 proteins in virus envelopes*. Virus captures assays were performed with microbeads conjugated with anti-human CD44 antibodies and HIV-1_LAI_ virus samples incubated with (virus + Ci) or without Ci extract (virus only). Levels of captured virus were determined by Gag-p24 ELISA of the eluate (left graph). Infectivity of the captured virus was determined with LC5-RIC cells (right graph). Bars represent the mean results of 3 replicates for each sample and error bars the standard deviation of the mean.

To verify whether antiviral Ci components target virus particles or host cells, we preincubated either cells or virus particles with Ci extract prior to infection. Preincubation of LC5-RIC cells with Ci extract had no effect on virus infection (Fig. 4A). In contrast, preincubation of virus particles with Ci strongly decreased infection, yielding infection levels even lower than those obtained by simultaneous addition of Ci extract and virus to cells (i.e. standard assay conditions). These results indicate that Ci extract directly affects virus particles.

To investigate whether Ci extract targets the HIV-1 envelope glycoproteins, we analysed whether treatment of virus particles with Ci extract affects capture of HIV-1 particles by heparin. HIV-1 binding to heparin is mediated by the viral envelope protein gp120 (reviewed in^33^). For heparin affinity chromatography, HIV-1 stocks were incubated with heparin-beads and eluted by washing with high salt buffer. Virus capture was determined by quantification of HIV-1 antigen (Gag-p24) associated with heparin beads and in the eluate. Virus antigen was not detected in the bead fraction or in the eluate of virus capture assays performed with Ci treated HIV-1, whereas virus antigen was readily detected for capture assays performed with untreated virus samples (Fig. 4B). These results indicate that Ci treatment blocks gp120-mediated binding to heparin.

Next we investigated whether Ci components also target cell-derived proteins in the viral envelope. The CD44 host protein is known to be incorporated into HIV envelopes (reviewed in^34^) and binding to anti-CD44 antibodies is used as a method to purify infectious virus particles. Therefore, we performed virus capture experiments with virus particles produced by CD44-positive cells (LC5-RIC) and magnetic microbeads conjugated to antibodies against CD44. Captured virus particles were quantified by HIV-1 Gag-p24 ELISA and levels of infectious virus particles determined with LC5-RIC reporter cells. As shown in Fig. 4C, virus particles were captured by the anti-CD44 microbeads both from Ci-treated and untreated virus samples. However, virus captured from Ci-treated samples showed strongly diminished infectivity, compared to virus captured from untreated samples.

These results indicate that Ci components block virus infectivity by targeting the virus envelope and inhibiting binding activity of the viral gp120 envelope protein but do not interact with the host-cell derived CD44 protein in the virus particle.

### Ci extract contains multiple components with anti-HIV activity

**Figure 5:**
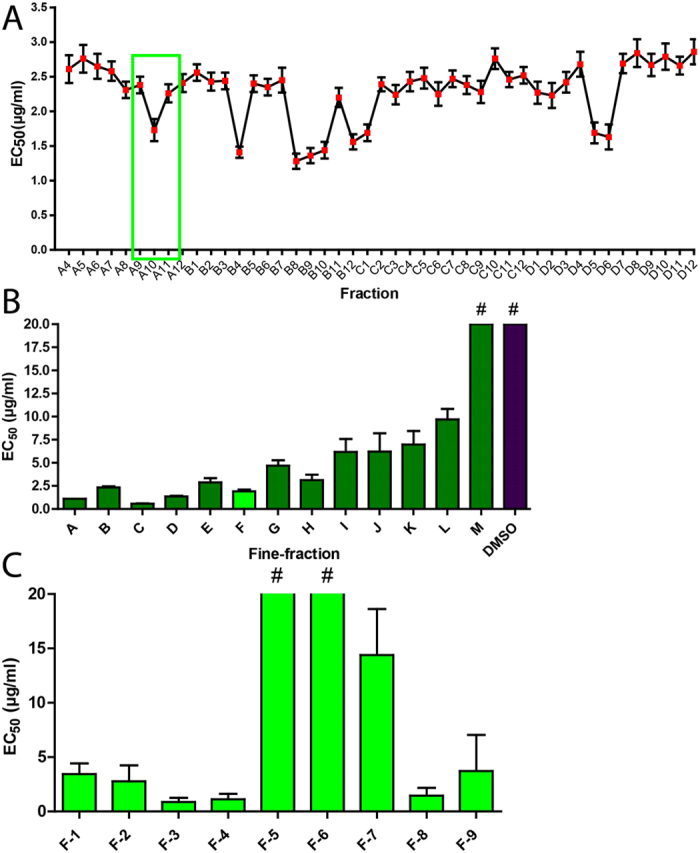
Profiling of anti-HIV activity during iterative fractionation of *Cistus incanus* (Ci) extract reveals multiple active compounds. (**A**) *Anti-HIV activity profile of 45 high-activity fractions obtained during initial separation of Ci extract*. The high-activity fractions A4-D12 were identified by single-dose anti-HIV screening of a set of 96 fractions generated in the initial separation step. Screening was performed with LC5-RIC cells and HIV-1_LAI_. For quantification of anti-HIV activity of each high-activity fraction, cells were exposed to the virus in the presence of different concentrations of the fraction, ranging from 100 μg/ml to 3.125 μg/ml (2-fold serial dilutions) and EC_50_ values determined. Symbols signify mean EC_50_ values of triplicate assays for each fraction and error bars the standard deviation of the mean. The frame marks an exemplary activity cluster, consisting of fractions A8 –A12. (**B**) *Anti-HIV activities of fine fractions derived from the exemplary activity cluster identified in A (fractions A8-A12).* The activity cluster was subjected to a second fractionation step, yielding fine fractions A-M, which were then assayed for anti-HIV activity. Bars indicate the mean values of 3 independent assays for each fraction and error bars the standard deviation of the mean. #: No antiviral activity detected in the measured concentrations. (**C**) *Anti HIV activities of high-resolution fractions derived from fine fraction F.* Fine fraction F was subjected to a third fractionation step, yielding high-resolution fractions F1 to F9 which were again assayed for anti-HIV activity. Bars indicate the mean values of 3 independent assays for each fraction and error bars the standard deviation of the mean. #: No antiviral activity detected at the highest concentration.

To gauge the complexity of compounds displaying anti-HIV activity in Ci extract, Ci extract was subjected to bioassay-guided fractionation. Ci extract was separated by a multi-step procedure validated for deconvolution of complex plant extracts^35^. First, a set of 96 fractions were prepared from Ci extract. These fractions were evaluated for anti-HIV activity by single-dose testing of all fractions (5 μg/ml), followed by analysis of dose-dependency of anti-HIV activity of 45 of the most active fractions. Several potential activity clusters were identified, of which one was exemplarily subjected to fine fractionation (Fig. 5A). Anti-HIV activity was demonstrated for the majority of fine fractions derived from this cluster, with several fine fractions yielding EC_50_ values below 5 μg/ml (Fig. 5B). Fine fraction F was chosen for further purification, based on high antiviral activity and favorable properties for structure elucidation, yielding high-resolution fractions F1-F9. Testing of antiviral activities of these high-resolution fractions (Fig. 5C) revealed two candidates (F-3, F-4) with high anti-HIV activity (EC_50_ < 1.1 μg/ml). Finally, HPLC analysis of high-resolution fraction F-4 yielded a single peak (99% purity).

These results demonstrate that Ci extract contains multiple anti-HIV compounds which can be separated by chemical extract deconvolution.

### No emergence of Ci-resistant HIV during long-term passaging

To investigate potential emergence of resistant viruses during long-term Ci treatment, we passaged HIV-1_LAI_ on LC5-RIC cells multiple times in the presence of Ci and tested the susceptibility of the passaged virus to Ci at regular intervals. 48 rounds of infection (=24 weeks) were performed and for each round of infection HIV-1 was incubated with Ci at concentrations corresponding to either EC_50_ or EC_90_ for 24 hours, followed by incubation without Ci for 2–3 days for virus outgrowth. Sensitivity of the outgrowth virus to inhibition by Ci extract was analysed after every second round of infection. This procedure was also performed without Ci as control.

**Figure 6:**
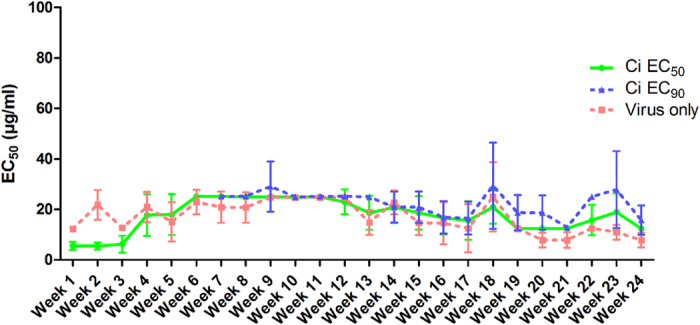
Lack of emergence of HIV-1 resistance during long-term virus passaging in the presence of *Cistus incanus* extract. Virus was passaged with or without Ci extract for 48 passages (i.e. 24 weeks) and monitored for sensitivity to inhibition by Ci extract every week. For each passage with Ci extract, LC5-RIC target cells were exposed to HIV-1_LAI_ virus in the presence of Ci extract for one day, followed by expansion of the escape virus for 2–3 days without Ci extract. Long-term virus passaging was performed with two concentrations of Ci extract, corresponding to EC_50_ (CiEC_50_ line graph) or EC_90_ (CiEC_90_ line graph) inhibitory concentrations against the initial virus inoculum. Passaging in the presence of the higher Ci extract concentration (CiEC_90_) was initiated with virus collected after 6 weeks of passaging with the lower Ci extract concentration. Virus was passaged in parallel without Ci extract as control (Virus only line graph). Virus collected from every second passage was evaluated for inhibition by Ci extract in infection assays with different concentrations of Ci extract (100 μg/ml to 3.125 μg/ml; two-fold serial dilutions) or without extract (=100% infection). Symbols represent the mean EC_50_ determined for each virus sample in three independent experiments, and the error bars the standard deviation of the mean.

As shown in Fig. 6, virus retained sensitivity to Ci treatment during the entire observation period at a stable level. Sensitivity of virus produced in the presence of Ci extract was comparable to sensitivity of virus produced in the absence of Ci treatment. These results indicate that long-term Ci treatment did not lead to emergence of Ci resistant viruses.

### Ci extract also targets Filovirus envelope proteins

**Figure 7:**
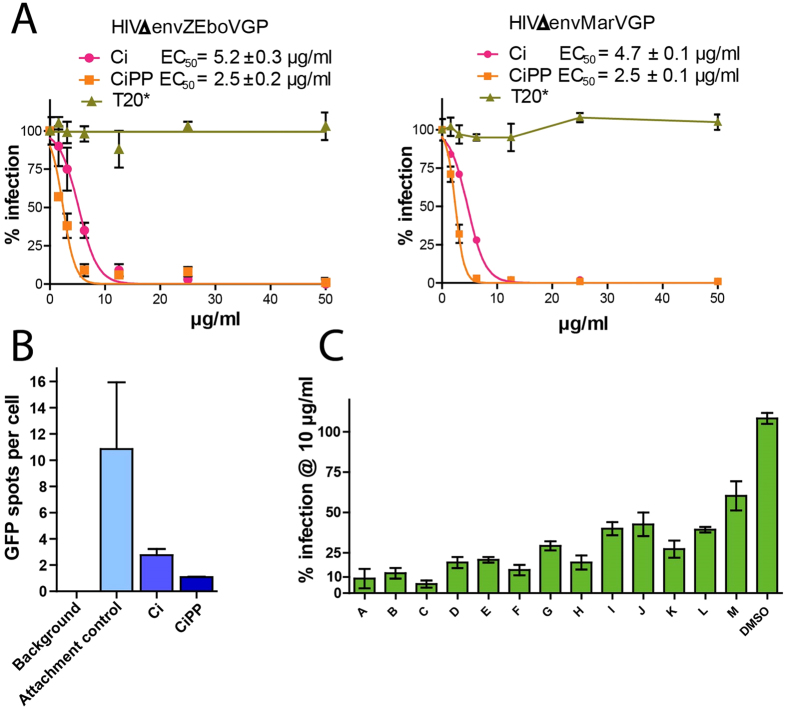
*Cistus incanus* (Ci) extract block Filovirus envelope proteins in pseudotyped lentivirus particles. Lentivirus particles pseudotyped with Filovirus envelope glycoproteins were produced by cells co-transfected with a plasmid containing an *env*-defective HIV-1 provirus (pBR-NL43-Gag-iGFP-Δenv) and plasmids encoding an envelope glycoprotein from Zaire Ebola virus (ZEBOV-GP) or Marburg Virus (MARV-GP). (**A**) *Ci extract and the polyphenol-enriched fraction of Ci extract (CiPP) inhibit infection of cells by virus particles pseudotyped with envelope proteins from Ebola virus (left panel) or Marburg virus (right panel).* LC5-RIC cells were exposed to pseudotyped lentivirus particles in the absence or in the presence of different concentrations of Ci extract, ranging from 50 μg/ml to 1.56 μg/ml (serial 2-fold dilutions). The HIV-1 fusion inhibitor T20 was assayed in parallel (500 nM to 15.63 nM). Dose-response curves are shown. EC_50_ is indicated for Ci and CiPP. For T20, the asterisk (*) signifies absence of antiviral activity against pseudotyped virus particles under test conditions. Symbols signify the mean values of triplicates, and the error bars the standard deviation of the mean. (**B**) *Ci and CiPP prevent primary attachment of pseudotyped virus particles to cells.* Attachment assays were performed with GFP-labelled virus particles pseudotyped with envelope glycoproteins for Ebola virus Zaire. GFP-signals associated with cells were quantified by spinning disc confocal microscopy and Volocity 6.2.1 imaging software. 60 cells in 4 images were analysed for each sample. The bars represent the mean values of GFP signals per cell and the error bars indicate the standard deviation of the mean. (**C**) *Fine fractions A-M generated during reiterative fractionation of Ci extract (see* Fig. 5B*) inhibit infection by pseudotyped virus particles.* Antiviral activity was tested with a fixed dose of Ci extract (10 μg/ml). Bars indicate the mean values of triplicate tests for each fraction and error bars the standard deviation of the mean.

This study shows that antiviral activity of Ci extract is directed against HIV-1 and HIV-2. Together with the previously reported inhibition of Influenza A virus^21^,^26^, these results strongly suggest that Ci extract has antiviral activity against multiple unrelated viruses. To further explore the spectrum of viruses inhibited by Ci, we investigated whether Ci extract also targets Ebola virus (EBOV) and Marburg virus (MARV) envelope glycoproteins. To this end, we generated pseudotyped HIV-1 virions carrying either Ebola virus Zaire subspecies (ZEBOV) or MARV glycoproteins^36^,^37^ instead of HIV-1 gp120 in their envelopes. As shown in Fig. 7A, whole Ci extract as well as Ci-derived polyphenols (CiPP) inhibited infection by the ZEBOV and MARV pseudotyped viruses (Fig. 7A). Antiviral activities against the pseudotyped virus proteins were comparable to those obtained for virions with native HIV-1 gp120 (Compare Figs 7A, 1 and 2). In contrast, the ZEBOV and MARV pseudotyped virions were not affected by the HIV-1 fusion inhibitor T20 up to concentrations of 500 nM, which is about 100-fold higher than the previously determined EC_50_ value for T20 in the EASY-HIT system^29^. Using GFP-labelled ZEBOV pseudotyped viruses, we demonstrated that Ci extract inhibited virus attachment to cells (Fig. 7B). Furthermore, we found that the fine fractions generated from cluster 1 and cluster 2 also inhibited infection of the ZEBOV pseudotyped virus, yielding a similar activity profile as that obtained with HIV-1 particles carrying native gp120 (Fig. 7C; compare with Fig. 5B).

Together these results demonstrate that antiviral activity of Ci extract is not limited to HIV-1 gp120 but also extends to Ebola virus and Marburg virus envelope glycoproteins, preventing these viral proteins from mediating infection.

## Discussion

There is an urgent need for potent, inexpensive and safe antiviral agents to enable treatment of acute and chronic life-threatening viral infections on a global scale. Various medicinal plants have been shown to inhibit major human viral pathogens like Herpesviruses, Dengue Viruses, Hepatitis B and C viruses and HIV^13^,^38^,^39^,^40^,^41^,^42^,^43^. Despite of these extensive studies, herbal products have not yet been advanced into broadly available, safe and efficacious medicinal agents for global treatment of viral infections^44^.

The standardized commercial Ci extract Cystus052^®^ has undergone clinical testing, displays many biological activities beneficial to human health, and was previously shown to inhibit Influenza A virus replication in cell culture^26^ and in an animal model^21^ (see also Introduction). Here we demonstrate potent antiviral effects of Ci extract against HIV and Filoviruses and show that Ci extract targets viral envelope components, blocking attachment of virus particles to cells.

Study of the anti-HIV activity of Ci extract (both whole extract and polyphenol-enriched fraction) with multiple HIV isolates revealed comparable antiviral efficacies against clinical isolates representing different major HIV genotypes, including HIV type 1 and type 2 and HIV-1 group O as well as group M. This finding was unexpected because of the high genetic diversity between HIV types 1 and 2 (over 50% amino acid diversity) and HIV-1 group M and O (over 35% amino acid diversity)^45^. Furthermore, HIV-2 and HIV-1 group O are less susceptible to several approved anti-HIV drugs than HIV-1 group M. Thus HIV-2 is inherently resistant to T20 (Efavirenz) and HIV-1 group O to several integrase and nonnucleoside reverse transcriptase inhibitors^46^,^47^,^48^,^49^. In addition, Ci extract prevented infection by virus particles containing Ebola or Marburg virus envelope proteins, demonstrating that antiviral activity of Ci extract also targets envelope components of Filoviruses. Together with previous reports of anti-Influenza A activity^21^,^26^, these results indicate that Ci extract displays broad-spectrum antiviral activity against multiple unrelated, major human viral pathogens (i.e. HIV, Marburg virus, Ebola virus, Influenza A virus).

Different experimental assays designed to investigate the mode of antiviral activity of Ci extract revealed that Ci extract prevents virus particles from interacting with the host cells. Treatment with Ci extract blocked delivery of the viral RNA into cells, demonstrating that Ci acts at the level of entry. Time-of-addition assays indicated that Ci treatment blocked entry of the virus at a very early stage, preceding fusion of viral and cellular membranes. Furthermore, Ci treatment inhibited attachment of GFP-labelled virus particles (HIV-1 and Ebola virus pseudotyped) to cells. Together these results confirm that Ci treatment prevents primary attachment of virus particles to cells, a mechanism-of-action conferring maximum protection of host cells against virus attack. In contrast, many other HIV and Ebola virus entry inhibitors act at a later step of the entry process and/or target host cellular components and do not protect cells from exposure to viral envelope proteins^50^,^51^,^52^. The collection of current clinically approved anti HIV drugs contains only two entry inhibitors^3^, i.e. fusion inhibitor T20 and the CCR5 co-receptor blocker Maraviroc. The majority of approved drugs target HIV enzymes i.e. protease, reverse transcriptase and a few integrase inhibitors. Since Ci extract inhibits HIV by a different mode-of-action it should be capable of inhibiting viruses with resistances to commonly used drugs. Indeed we showed that Ci extract effectively blocked *in vitro* replication of a multi-resistant HIV-1 isolate (HIV-1_V13-03413_) with mutations conferring resistance to all approved protease and reverse transcriptase inhibitory drugs.

This study also provides evidence that antiviral components of Ci extract target viral envelope proteins and show selectivity for viral over cellular components. Pre-incubation assays confirmed that antiviral components of Ci extract specifically target virus particles but are not retained by host cells. Further support for virus-selectivity of antiviral activity of Ci extract comes from the HIV-1 virus capture assays performed in this study, which show that Ci extract inhibits gp120-mediated binding of virus particles to heparin, but does not prevent binding of the host-cell derived CD44 protein with its antibody ligand. Selective blocking of gp120 but not CD44 protein in virus particles suggests that components of Ci extract specifically mask gp120 on viral envelopes rather than unspecifically enwrapping entire virus particles.

Not only HIV-1^33^ but numerous other unrelated viruses use heparin and heparan sulfate moieties of proteoglycans for primary attachment to cells. In addition to Filoviruses, these include Herpes viruses and Hepatitis B and C viruses^53^,^54^,^55^,^56^. Our finding that Ci extract blocks binding of viral components to cell moieties used by numerous viruses for attachment provides further support for broad activity of Ci extract against different viruses. Recently, a single-molecule natural product from green tea was also reported to inhibit primary attachment of different virions to heparan sulfate glycans^57^, providing evidence for the occurrence of broad-spectrum natural products that block virus attachment to cells. With respect to HIV, targeting of gp120 suggests that Ci extract may also protect cells from harmful consequences of gp120-cell interactions that go beyond HIV entry. These include induction of aberrant signaling events^58^,^59^, apoptosis of T-cells^60^ and neuronal damage^61^,^62^.

Cell culture experiments designed to investigate the propensity of Ci extract to induce virus resistance revealed no outgrowth of Ci-resistant virus during 24 weeks (=168 days) of passaging of HIV-1 in the presence of Ci extract. In contrast, the risk of virus resistance was reported to be much higher for the single-compound attachment inhibitor Fostemsavir (BMS-663068)^63^. Thus, 14 days of selection in cell culture were sufficient to generate Fostemsavir-resistant HIV-1_LAI_ viruses and a single amino acid substitution in HIV-1_LAI_ Env was sufficient for resistance. Bioassay-guided fractionation studies indicate the presence of multiple antiviral compounds in Ci extract. This combination of active molecules is presumably responsible for the low risk of the emergence of resistant viruses during Ci treatment, compared to treatment with single-compound antiretroviral drugs.

This study demonstrates potent and broad antiviral activity of Ci extract under cell culture conditions. Furthermore, clinical studies have shown a favorable safety profile for Ci extract *in vivo*^27^,^28^. Although these observations support further exploration of the antiviral activities of Ci extract, it is important to point out that we do not advocate the use of Ci extract as drug on the basis of the results presented in this study. Evaluation of the potential of Ci extract for *in vivo* antiviral therapeutic applications requires extensive future investigations. An important step in this direction will be the identification of individual antiviral agents from Ci extract and analysis of their pharmacokinetic properties. Another avenue for exploration of *in vivo* application of Ci extract is its potential use as topical microbicides to prevent sexual transmission of HIV transmission^64^. The resistance of antiviral activity to low pH and high temperatures found in this study are further encouraging properties of Ci extract supporting its exploration for topical applications.

Finally, Ci extract may also provide a source for mining of novel antiviral agents. Thus, natural products are regaining popularity for drug discovery because they overcome various restrictions of synthetic libraries, including limited chemical diversity^65^. Furthermore, natural products proved to be very successful leads for drug development in the past, with 34% of drugs approved by the FDA from 1981 to 2010 being either based on or derived from natural products^66^. We show that antiviral activity can be isolated from Ci extract by enrichment of polyphenolic compounds and is retained throughout numerous sequential fractionation steps. This indicates that active agents with reduced complexity can be produced from Ci extract. Identification of single active compounds from complex or simplified Ci extract may yield leads for novel antiviral agents, as well as provide signature molecules for monitoring of pharmacological properties of Ci-derived antiviral agents in clinical studies.

## Conclusion

Ci extract combines broad antiviral activity with low risk of virus resistance. Its mechanism-of-action involves blocking of primary virus attachment to cells by selective targeting of the viral envelope glycoproteins. In contrast, many other natural products with broad-spectrum antiviral activity target host cell components^67^. Our results demonstrate that multiple antiviral components within the Ci extract block virus attachment to the host cell. Altogether, the broad and potent *in vitro* antiviral effects of Ci extract demonstrated in this study support the further exploration of Ci extract and its components for novel antiviral approaches.

## Materials and Methods

### Viruses and virus production

Production and quantification of HIV-1 stocks was carried out as described in^15^,^29^. Briefly, stocks of HIV-1 laboratory isolates were produced by transfecting HEK 293 T cells with one of the following proviral plasmids obtained through the NIH AIDS Research and Reference Reagent Program, Division of AIDS, NIAID, NIH: pLAI.2 (from Dr. Keith Peden^68^), pNL(AD8) (from Dr. Eric O. Freed^69^). GFP-labelled viruses were produced by transfection with pBR-NL43-Gag-iGFP^32^. Virions with CD44 in the envelope were generated by infecting LC5-RIC cells (positive for surface CD44) with HIV-1_LAI_ virus. Virus-containing cell culture supernatants were harvested 72 h post transfection/ infection and stored at −80 °C. Virus levels in virus stocks were quantified by Gag-p24 antigen enzyme-linked immunosorbent assay (ELISA) (Advanced Bioscience Laboratories, Maryland) and by titration on LC5-RIC cells as described in^29^.

Primary clinical HIV-isolates HIV-1M_MVP899-87_, HIV-1O_MVP5180-91_ HIV-2_MVP10668-93_ and HIV-1_V13-03413B_ were obtained as described in^70^. The multiresistant isolate HIV-1_V13-03413B_ was obtained from a patient with virologic treatment failure and shown by sequence analysis to contain multiple drug resistance mutations in the regions encoding the reverse transcriptase (RT) and protease (PR). RT: T69I, V75I, L100I, K103N, Y115F, V118I, Q151M, V179L, M184V, L210F, K219E, H221Y; PR: L10I, V11I, K20R, V32I, L33F, E35D, M46I, I54L, Q58E, A71V, G73T, V82A, I84V, L90M. For production of clinical isolates, frozen stocks of HIV-infected H9 cells in virus containing cell-culture supernatant were thawed and cultured for 6 cell passages. Every three to four days, about half of the infected cell population was replaced with uninfected H9 cells and about 60–70% of the culture supernatant of the infected cell population replaced with fresh medium. Infectious virus levels in culture supernatants were titrated with LC5-RIC reporter cells as described in^29^. HIV inhibition experiments were performed with volumes of virus inoculum yielding an at least 100-fold increase of relative fluorescent signal intensities of LC5-RIC reporter cultures in the absence of inhibitors.

For the production of Ebola or Marburg virus glycoprotein pseudotyped virus, HEK293T cells were co-transfected with the HIV backbone plasmid pBR-NL43-Gag-iGFP-Δenv^71^ and with the glycoprotein expression plasmids pCAGGS-ZEBOV-V5^36^ or pCAGGS-Marv-V5^37^ and pseudotyped virus particles were harvested 72 h post transfection and stored at −80 °C.

### Cells and cell culture

Adherently growing cell lines (HEK293T, LC5-derivates) were cultured in Dulbecco’s Modified Eagle’s Medium (DMEM; Gibco® Life Technologies, USA) supplemented with 10% FCS and 1% antibiotic-antimycotic solution (Gibco® Life Technologies, USA). H9 cells were cultured in suspension, in Very-Low-Endotoxin Roswell Park Memorial Institute-1640 Medium (VLE-RPMI 1640; Gibco® Life Technologies, USA), supplemented with 10% FCS and 1% antibiotic-antimycotic solution (Gibco® Life Technologies, USA). All cells were maintained under standard conditions (37 °C; 5% CO_2_).

The human embryonal kidney cell line HEK293T (ATCC®-Number CRL-11268) and the human T-lymphoma cell line H9 (ATCC®-Number HTB-176) were obtained through ATCC. The LC5 cell line is derived from HeLa cells. The generation, characteristics and culture of LC5-CD4, LC5-RIC and LC5-RIC-R5 are described in detail in^15^,^29^,^72^. Briefly, LC5-CD4 cells contain stably integrated sequences for expression of human CD4 and neomycin resistance. LC5-RIC cells were derived from LC5-CD4 cells by stable integration of a DsRed-reporter gene under the control of HIV-1 Tat and Rev and sequences for hygromycin B resistance. LC5-RIC-R5 cells were derived from LC5-RIC cells by stable integration of a human CCR5-encoding cDNA and sequences for puromycin resistance. LC5-derived cell lines were cultured with the appropriate antibiotics at regular intervals.

Peripheral blood mononuclear cells (PBMC) were isolated from buffy-coats by Ficoll (Biochrom AG, Germany) gradient centrifugation as described before in Fuss *et al.*^73^. Cells were then washed and treated with ammonium chloride potassium (ACK) buffer (Gibco® Life technologies, USA) to remove residual platelets and erythrocytes. The cells were immediately cultivated in VLE-RPMI 1640 medium (Biochrom AG, Germany) supplemented with 10% FCS and 1% antibiotic-antimycotic solution (Gibco® Life technologies, USA) overnight and 20 U/ml hIL-2 (Roche, Germany) and 1 μg/ml phytohemagglutinin (PHA) (Biochrom AG, Germany) were added to the cells for stimulation. The cells were stimulated for three days before they were used for infection.

### *Cistus incanus* extracts and fractions

The main source of Ci extract used for antiviral testing was the medicinal product CYSTUS052® (Dr. Pandalis Urheimische Medizin GmbH und Co. KG, Germany). In addition antiviral activity was confirmed in Ci extract generated from dried *Cistus incanus* herbs (Cistus incanus Bio Tee, Dr. Pandalis Urheimische Medizin GmbH und Co. KG, Germany) and from fresh plants (Cystus®, *Cistus incanus ssp. Tauricus;* Rühlemann’s Kräuter und Duftpflanzen, Horstedt, Germany) by boiling the plant material in water for 1 hour. All Ci extracts were sterile-filtered (0.45 μm syringe-filter Millex®-HV, Merck Millipore, Darmstadt, Germany), dried in an Eppendorf Vacuum Concentrator, the weight of the dry mass determined and the dry mass stored at −20 °C until use. For testing, the dry mass was dissolved in cell culture medium, with stock solutions typically containing 10 mg dry mass per ml.

Polyphenols were enriched from Ci extract derived from CYSTUS052® as described in Helfer *et al.*^15^. Briefly, polyphenols were adsorbed to polyvinylpyrrolidone (PVPP, Sigma Aldrich) and eluted with 0.5 N NaOH. Eluates were equilibrated to pH 6 with HCl and purified by solid-phase extraction (SPE) with a C18 SPE-column (Bond Elut C18, Agilent Technologies, California, USA).

Separation of Ci extract for extract deconvolution was performed by Bicoll GmbH, using proprietary technology for deconvolution of plant extracts.

### Cell-based assays for evaluation of antiviral activities

#### Inhibition of Infection Assays

The standard assay format for testing inhibition of virus infection by antiviral agents is described in Kremb *et al.*^15^,^29^. Briefly, LC5-RIC reporter cells were seeded in 96-well plates at a density of 1 × 10^4^ cells per well in 100 μl culture medium. After 24 hours, test compounds and appropriate volumes of virus inoculum resulting in 10 pg Gag-p24/cell or ≥100 relative fluorescence intensity increasing units per culture were added to the cells. Plates were incubated for 48 hours and fluorescent signals of cultures were measured with a Tecan Infinite M200 plate reader (Tecan, Crailsheim, Germany) at excitation/emission wavelengths of 552/596 nm. Test compounds were assayed in at least 6 concentrations, generated by 2-fold serial dilutions. Controls consisted of cultures exposed to the virus without test compound (=100% infection) and cultures lacking virus and compound (=background). Each compound concentration was tested in at least triplicate wells and controls in six wells. Fluorescent signals were corrected for background signals and fluorescent signals of compound-treated cultures normalized to those of untreated, virus-exposed cultures (=100% infection). The antiviral potency of the test compound was expressed as the half maximal effective concentration (EC_50_), which signifies the concentration calculated to yield 50% inhibition of infection.

Inhibition of infection of primary human target cells was tested with stimulated PBMCs seeded in 96-well plates (1 × 10^5^ cells per well) in culture medium containing hIL-2 and PHA (see above) and cultured for 24 hours. Virus inoculum (10 pg Gag-p24/cell) and test compounds were added to the cultures (120 μl total culture volume). Test compounds were assayed at different concentrations (see above) in at least 3 replicate wells for each concentration and controls in 6 replicate wells. After 48 hours, 20 μl of culture supernatant from each well was transferred to LC5-RIC reporter cells and fluorescent signals of reporter cultures measured 72 hours later.

#### Time-of-addition (TOA) assays

TOA assays were performed with LC5-RIC cells in 96-well plates. The assay format was similar to that used for determining inhibitor potencies (see above), including positive and negative controls, with a few modifications. Inhibitors were added to the wells either simultaneously with the HIV-1_LAI_ virus inoculum (defined as time point 0) or at various later time points and fluorescent signals of cultures determined 48 hours after virus addition. In addition to Ci extract, T20 and Efavirenz were used as reference inhibitors (both obtained through the NIH AIDS Research and Reference Reagent Program, Division of AIDS, NIAID, NIH). To ensure full inhibition, inhibitor concentrations used for treatment exceeded 5 × EC_50_, i.e. 100 μg/ml Ci extract, 100 nM Efavirenz and 500 nM T20.

*Preincubation assays* were performed in a standard infection assay format (see above), using different concentrations of Ci extract ranging from 100 μg/ml to 3.12 μg/ml (2-fold serial dilutions). For preincubation of cells with Ci extract, LC5-RIC cultures were incubated with Ci extract for 3 hours, cells were washed once with PBS and virus (HIV-1_LAI_) added in fresh medium to cells for infection. For preincubation of virus inoculum with Ci extract, virus inoculum was preincubated with the appropriate dilutions of Ci extract for three hours and the virus-Ci mixture transferred to LC5-RIC cells for infection. In a parallel plate, cells were exposed simultaneously to Ci extract and virus inoculum, corresponding to the conditions used in the standard inhibition of infection assay.

*Virus attachment assays* were performed as described in Helfer *et al.*^15^. Briefly, LC5-RIC cells were seeded on glass cover slips (24 × 24 mm) in 12-well plates and were incubated with GFP-labelled HIV-1 virions or pseudotyped virus particles (1 ml culture supernatant of HIV-1 or pseudotyped virus producing cells per well) either in the presence of 100 μg/ml whole Ci extract (Ci) or Ci polyphenol fraction (CiPP). As positive control for virus attachment, cells were exposed to virus without Ci extract. The background control consisted of cells cultured without virus and Ci extract. Attachment of HIV-1 virions was assayed in the presence of 50 nM T20 fusion inhibitor to block virus entry into cells. After incubation for 4 hours, cells were washed with PBS, fixed with 2% paraformaldehyde for 30 min, washed again 3 times and incubated in DAPI-staining solution (1:10 000) for 15 min to stain intracellular DNA. Coverslips were fixed onto glass slides and samples analysed by spinning disk confocal microscopy, using a Nikon TiE equipped with the Perkin Elmer UltraView Vox system (exposure times GFP: 100 mS, DAPI: various; Brightfield: various). Volocity 6.2.1. software was used to quantify virus particles (=GFP spots; defined as all pixels within a radius of 1 μm around the brightest spot, intensity threshold 755). DAPI-positive cells were counted manually.

### Virus capture assays

Virus capture assays based on binding of host-derived CD44 in viral envelopes to immobilized ligand were performed with magnetic microbeads conjugated with anti-human CD44 antibodies (μMACS VitalVirus HIV Isolation Kit, Miltenyi Biotec, Germany) and HIV-1_LAI_ produced by LC5-RIC cells (CD44 positive), according to manufacturer’s instructions. Culture supernatants of HIV-1_LAI_ infected LC5-RIC cells were centrifuged at 3,000 *g* to remove cell-debris. Samples (1 ml) containing equivalent amounts of HIV-1 Gag-p24 (59 ng/μl) and 200 μg/ml Ci extract or DMEM were incubated under standard cell culture conditions. After 2 hours, anti-CD44 microbeads (250 μl) were added to the samples and incubation continued for 30 minutes. The virus/microbead mixture was loaded onto a μMACS column within a magnetic field and afterwards columns were washed 3 times. For elution of virus, columns were removed from the magnetic field, and virus particles were eluted with 300 μl DMEM. Virus levels in eluate, the flow-through and wash fractions were determined by Gag-p24 antigen ELISA and by titration of 20-2.5 μl in a 2-fold serial dilution in a standard infection assay with LC5-RIC cells.

The virus capture assays based on binding of viral gp120 envelope protein to heparin-beads were performed with a procedure modified from Salvador *et al.*^54^. For preparation of virus samples, 10 ml culture supernatant of HIV-1_LAI_ producing cells were concentrated by ultracentrifugation with Amicon® Ultra −15 100 k Centrifugal Filter Units (Merck Millipore, Darmstadt, Germany) and the retentate taken up in 10 ml binding buffer (10 mM sodium phosphate in PBS). 1 ml virus solution was either incubated with 150 μg/ml Ci or the respective amount of PBS for 3 hours. Heparin-beads were extracted from HiTrap™ Heparin HP, 1 ml columns (GE Healthcare; Germany), washed twice with PBS and resuspended in 5 ml binding buffer. 200 μl bead-solution was added to 600 μl virus-solution (+ or −Ci) and the virus-bead mixtures were incubated for 10 minutes at RT. Beads were then pelleted at 900 g for 3 min, the supernatant was discarded and the bead-pellet washed twice with 1 ml PBS. A sample of the bead-pellet (100 μl) was removed for evaluation of virus binding to beads. The remaining bead-pellet was resuspended in elution buffer (10 mM sodium phosphate in PBS; 2 M NaCl), beads were removed by centrifugation and the supernatant (=eluate) was collected. Bead-pellets and eluates were stored at −20 °C and evaluated for the presence of HIV-1 by Gag-p24 antigen ELISA.

### Evaluation of HIV genomic RNA delivery to cells by qRT-PCR

Each experiment consisted of 3 samples of virus-exposed cultures and one background sample of cultures not exposed to the virus. All samples were tested in duplicate. Virus-exposure was performed either in the presence of whole Ci extract (Ci) or Ci polyphenol fraction (CiPP) or without Ci extract to assess maximum virus RNA delivery. LC5-CD4 cells were seeded in 6-well plates (4*10^5^ cells per well), incubated for 24 hours and HIV-1_LAI_ (10 pg Gag-p24/cell) added, either together with (100 μg/ml) or without Ci extract. All cultures also contained 50 nM Efavirenz (obtained through the NIH AIDS Research and Reference Reagent Program, Division of AIDS, NIAID, NIH), to prevent reverse transcription of delivered viral RNA.

Four hours after virus addition, the cells were extensively washed, harvested and RNA was isolated using the RNeasy-kit (Quiagen, Netherlands) according to the manufacturer’s instructions. HIV-1 RNA levels were quantified relative to transcript levels of the RNA polymerase II (RPII) housekeeping gene by qPCR. For this purpose, cDNA was generated from RNA by reverse transcription with random hexamers, using the Superscript®III First-Strand Synthesis System (Life Technologies, USA) according to the manufacturer’s protocol. qPCR was performed with FastStart DNA Master SYBR Green I-Kit, the Roche Light Cycler 1.5 instrument and LightCycler 480 Software, Version 1.5 (standard LightCycler protocol). The following primers were used for amplification of HIV-1 sequences: HIV-forward primer 5′-CCA GTC ACA CCT CAG GTA CCT TTA AGA CC-3′ and HIV -reverse primer 5′-GTG TGT GGT AGA TCC ACA GAT CAA GG-3′. RPII sequences were amplified with the following primers RPIIs 5′-GCA CCA CGT CCA ATG ACA-3′ and RPIIas 5′-GTG CGG CTG CTT CCA TAA-3′. The data was analysed with the second derivative maximum method and relative expression ratios were calculated by the 2^–ΔΔCT^ method^74^, with ΔCT representing [CT (HIV target) - CT (RPII reference]. ΔΔCT signifies ΔCT virus-exposed samples minus ΔCT of background samples (no virus).

### Resistance Assay

HIV-1_LAI_ was passaged 48 times (24 weeks) in LC5-RIC cells with or without Ci extract in 6-well plates. For each passage, cells were exposed to virus in the presence of Ci extract for 24 hours of infection. The medium was then exchanged for fresh medium and cultures were incubated for 48 to 72 hours for virus production. Culture supernatants were collected and 500 μl used as inoculum for the next virus passage. Treatment with Ci extract was performed with 8.06 μg/ml or 15.5 μg/ml Ci extract, corresponding to EC_50_ and EC_90_, respectively (determined with initial HIV-1 inoculum). Passaging was performed in parallel in the absence of Ci extract as control.

Every second passage, sensitivity of virus to inhibition by Ci extract was determined by standard infection assays, using 5 μl of culture supernatants and concentrations of Ci extract ranging from 100 to 3,125 μg/ml to determine EC_50_ values.

### Cell viability assay

Compound effects on cell viability were monitored by MTT (3-(4,5-dimethylthiazol-2-yl)-2,5-diphenyl tetrazolium bromide) assay, which measures the reduction of yellow MTT to purple formazan by mitochondrial enzymes^75^. The MTT assay was performed as described in Kremb *et al.*^29^.

### Statistical analysis

EC_50_/CC_50_ values were calculated with GraphPad Prism v5, using the equation for sigmoidal dose-response with variable slope and constraints set to 100 for top and 0 for bottom.

## Additional Information

**How to cite this article**: Rebensburg, S. *et al.* Potent *in vitro* antiviral activity of *Cistus incanus* extract against HIV and Filoviruses targets viral envelope proteins. *Sci. Rep.* **6**, 20394; doi: 10.1038/srep20394 (2016).

## Supplementary Material

Supplementary Information
