# Supplementary material for: Potent in vitro antiviral activity of Cistus incanus extract against HIV and Filoviruses targets viral envelope proteins
Source: Sci Rep. 2016 Feb 2;6:20394. doi: 10.1038/srep20394 (PMC4735868; doi:10.1038/srep20394)
Supplement: Supplementary Information [file srep20394-s1.doc]

**Potent *in vitro* antiviral activity of *Cistus incanus* extract against HIV and Filoviruses targets viral envelope proteins**

**†Rebensburg S**. ^1^, †**Helfer M. ^1^, Schneider M. ^1^, Koppensteiner H. ^1^, Eberle J. ³, Schindler M. ^1,2^, Gürtler L. ^3^, Brack-Werner R.^1*^**

***Supplementary Information***

**Supplementary Figure S1:**

**Aqueous extracts of *Cistus incanus* (Ci) from different sources display anti-HIV activity.**

***
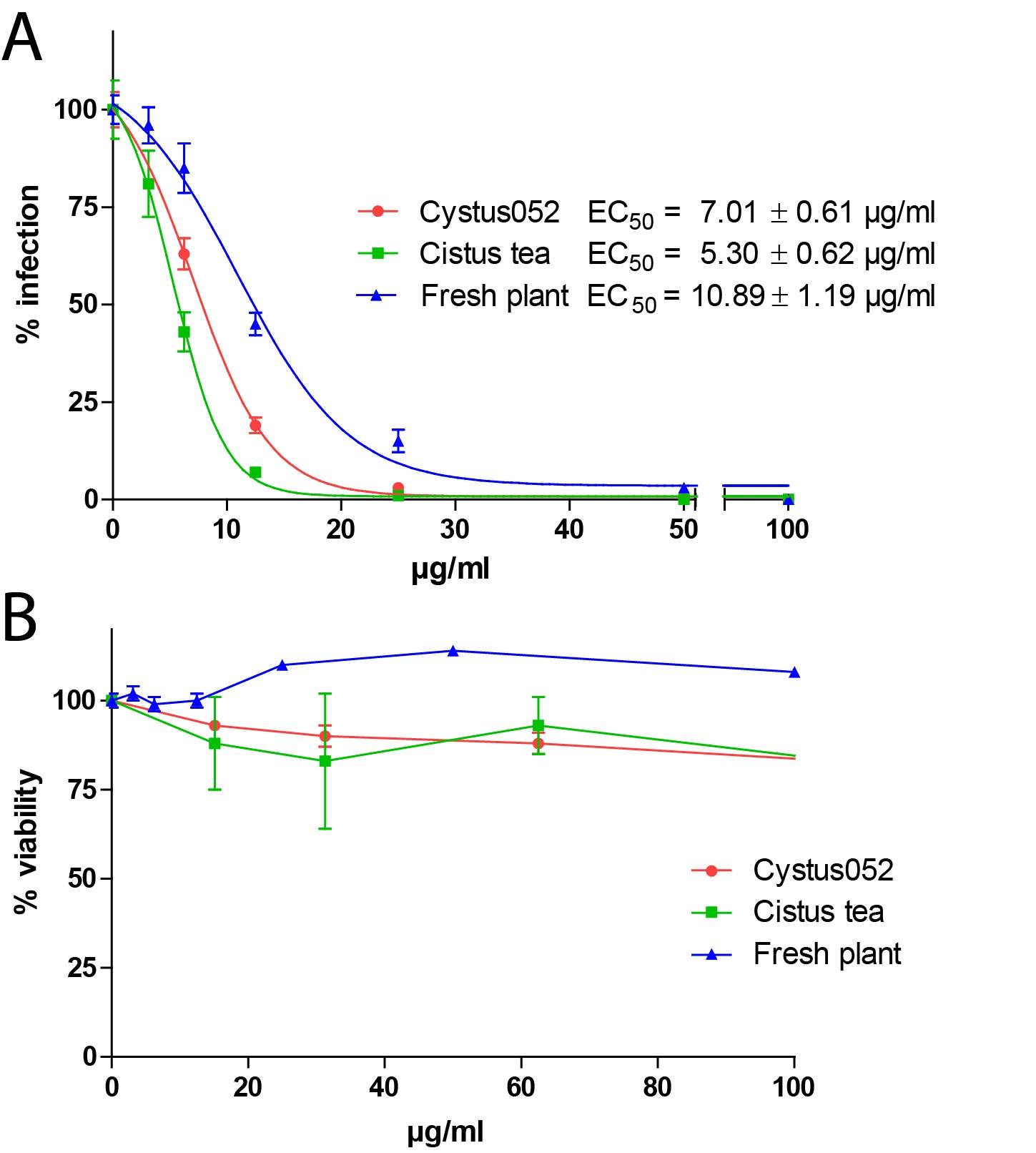
***

(**A***) Anti-HIV activities of Ci extract from different sources*. Ci extracts examined consisted of the aqueous commercial Ci medical product CYSTUS052® Decoction (Cistus052) and extracts produced by boiling either commercial dried Ci herbs (Cistus tea) or minced fresh plants (Fresh plant; n=3) with water. All extracts were sterile-filtered, dried by evaporating in an Eppendorf vacuum concentrator and the dry mass dissolved in cell culture medium to generate Ci stock solutions of 10 mg dry mass/ml.

Anti-HIV activity of the extracts was evaluated with HIV-1_LAI_ and LC5-RIC cells under standard infection assay conditions. Dose-response curves were generated with 6 concentrations of Ci extract between 100µg/ml and 3.12µg/ml (2-fold serial dilutions). Each Ci concentration was tested in triplicate cultures. Symbols signify mean values and error bars the standard deviation of the mean.

(**B**) *Effects of Ci extracts on viability of cells.* MTT assay was used to confirm viability of cells in cultures during antiviral testing. Viability of cells exposed to HIV-1 and Ci extract were related to cell viability in cultures exposed to the virus without Ci extract (= 100%). Symbols represent mean values of triplicate cultures and error bars the standard deviation of the mean.

**Supplementary Figure S2:**

**Comparison of the effects of *Cistus incanus* (Ci) whole extract and the polyphenol-enriched fraction of Ci (CiPP) on viability of peripheral blood mononuclear cells (PBMC).**


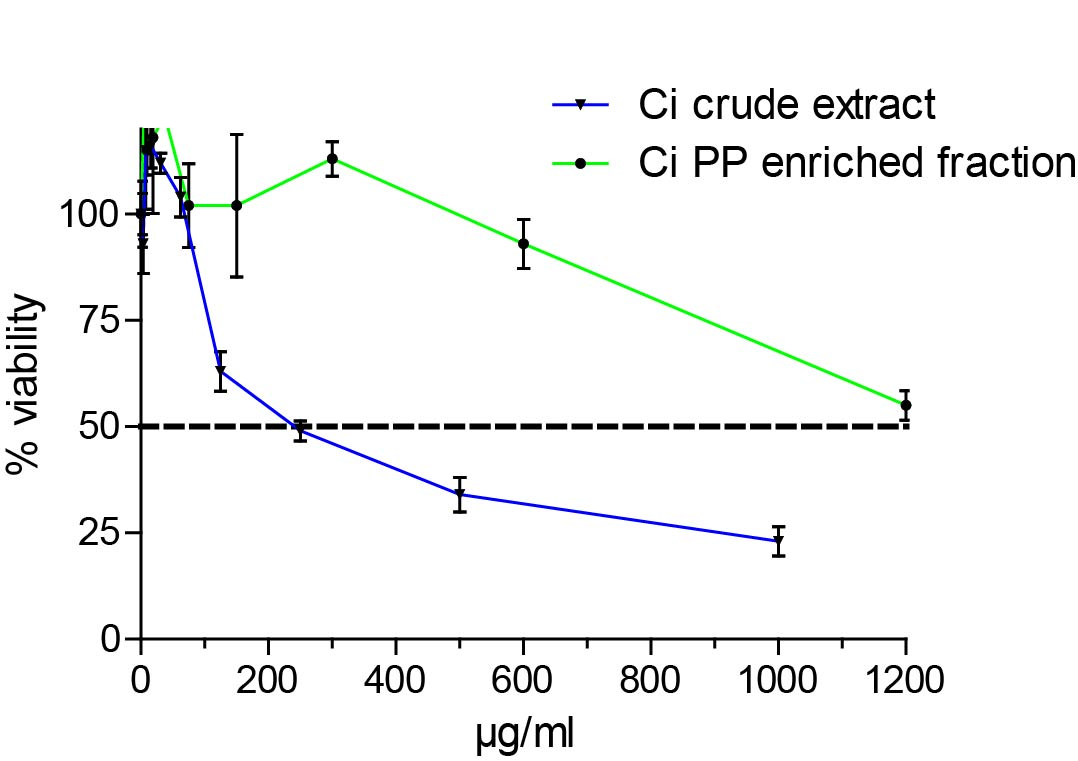


Effects of Ci extract on viability of PBMCs. PBMCs from one donor were isolated as explained above and stimulated for 3 days with 20U/ml hIL-2 and 1µg/ml PHA. After stimulation the cells were seeded in 96-well plates (1 x 10^5^ cells per well) and the following day HIV-1_LAI_ and compounds were added for 48h. The Ci whole extract was tested in 2 fold serial dilutions from 1000µg/ml to 31.25µg/ml and the CiPP fraction was tested from 1200 µg/ml to 37.5µg/ml. MTT assay was used to confirm viability of cells in cultures during antiviral testing. Viability of cells exposed to HIV-1_LAI_ and Ci extract were related to cell viability in cultures exposed to the virus without Ci extract (= 100%). Symbols signify mean values of triplicate cultures and error bars the standard deviation of the mean.
